# The Value of PET/CT in Particle Therapy Planning of Various Tumors with SSTR2 Receptor Expression: Comparative Interobserver Study

**DOI:** 10.3390/cancers16101877

**Published:** 2024-05-15

**Authors:** Carola Lütgendorf-Caucig, Patricia Wieland, Eugen Hug, Birgit Flechl, Slavisa Tubin, Razvan Galalae, Petra Georg, Piero Fossati, Marta Mumot, Semi Harrabi, Irina Pradler, Maciej J. Pelak

**Affiliations:** 1 MedAustron Ion Therapy Center, 2700 Wiener Neustadt, Austria; 2 Radioonkologie, Department of Radiation Oncology, Heidelberg University Hospital, 69120 Heidelberg, Germany; 3 Heidelberg Ion-Beam Therapy Center (HIT), Department of Radiation Oncology, Heidelberg University Hospital, 69120 Heidelberg, Germany; 4 Faculty of Human Medicine, Karl Landsteiner University for Health Sciences, 3500 Krems, Austria; 5 Medizinische Fakultät, Christian-Albrechts-Universität zu Kiel, 24118 Kiel, Germany; 6 Klinische Abteilung für Strahlentherapie—Radioonkologie, Universitätsklinikum Krems, 3500 Krems, Austria; 7 Division of Radiation Oncology, Karl Landsteiner University for Health Sciences, 3500 Krems, Austria; 8 Universitätsklinik für Radiotherapie und Radio-Onkologie, Landeskrankenhaus Salzburg, 5200 Salzburg, Austria

**Keywords:** PET/CT, DOTA, glomus tympanicum tumor, meningioma, PitNET, proton therapy, contouring, interobserver variability

## Abstract

**Simple Summary:**

Multiple tumor types feature frequent overexpression of somatostatin receptor type 2 (SSTR2). In addition to its well-established role in determining the primary diagnosis, a PET/CT can be helpful in the radiation treatment of these tumors. It has the potential to improve recognition of the tumor burden compared to CT or MRI alone. In a blinded comparative interobserver study, we anonymized 47 patients with various tumors with SSTR2 expression and instructed four radiation oncologists to independently contour the macroscopic tumor volume using MRI alone and subsequently with the addition of DOTA-conjugated PET/CT. This study showed that for meningioma and skull base paraganglioma (SBPGL), there was a better consensus and certainty between the observers, with the opposite trend for pituitary neuroendocrine tumors (PitNET). For PitNET and meningioma, the addition of PET/CT led to higher sensitivity between the observers compared to CT and MRI alone, suggesting the benefits of integrating DOTA-conjugated PET/CT for target definition.

**Abstract:**

The overexpression of somatostatin receptor type 2 (SSTR2) is a property of various tumor types. Hybrid imaging utilizing [^68^Ga]1,4,7,10-tetraazacyclododecane-1,4,7,10-tetra-acetic acid (DOTA) may improve the differentiation between tumor and healthy tissue. We conducted an experimental study on 47 anonymized patient cases including 30 meningiomas, 12 PitNET and 5 SBPGL. Four independent observers were instructed to contour the macroscopic tumor volume on planning MRI and then reassess their volumes with the additional information from DOTA-PET/CT. The conformity between observers and reference volumes was assessed. In total, 46 cases (97.9%) were DOTA-avid and included in the final analysis. In eight cases, PET/CT additional tumor volume was identified that was not detected by MRI; these PET/CT findings were potentially critical for the treatment plan in four cases. For meningiomas, the interobserver and observer to reference volume conformity indices were higher with PET/CT. For PitNET, the volumes had higher conformity between observers with MRI. With regard to SBGDL, no significant trend towards conformity with the addition of PET/CT information was observed. DOTA PET/CT supports accurate tumor recognition in meningioma and PitNET and is recommended in SSTR2-expressing tumors planned for treatment with highly conformal radiation.

## 1. Introduction

The management of benign tumors of the base of the skull is frequently challenging due to their proximity to critical structures such as the brain parenchyma, cranial nerves and major blood vessels. These tumors can originate from various tissues including meninx, neural crest cells of paraganglia or glandular tissue. The most common types include meningiomas, skull base paragangliomas (SBPGL) and pituitary neuroendocrine tumor (PitNET)/pituitary adenomas. Cases with asymptomatic and indolent courses are often considered for the watch-and-wait approach, whereas local therapy is indicated in case of radiologic progression and/or a symptomatic course, in particular involving mass effect on brain parenchyma, compression of neurological structures located in the skull base or, in the case of pituitary adenomas, oversecretion of various hormones.

For cases with an indication for local treatment, maximum safe surgical resection is widely recognized as the standard of care. Radiotherapy, either primary or postoperative following incomplete resection, is indicated if further surgery is associated with unacceptable risk of postoperative complications including functional and neurological sequelae. The goals of radiotherapy in this context include tumor control, the preservation of neurological function and the prevention of recurrence [1,2,3]. Proton therapy allows for better precision in delivering radiation to the tumor thanks to its high dose conformality and sharp dose fall-off outside of the contoured volume. This leads to minimizing radiation exposure to surrounding healthy tissues and nearby organs at risk. Hence, proton therapy offers an advantage in reducing the risk of acute and long-term side effects associated with radiation therapy while maintaining an excellent local control rate. This is particularly relevant for benign skull base tumors, as the patients are commonly seen to have very high overall survival rates [4].

Because of the remarkably high dose gradient of modern proton beam therapy (PBT), correct and accurate target definition and organ-at-risk contouring is crucial. Current imaging modalities for both target definition and organ-at-risk contouring in radiotherapy mainly comprise CT and MRI scans. However, CT and MRI scans are both principally structural imaging modalities and are, therefore, subject to certain limitations. On one side, contrast uptake can be attributed not only to tumor tissue but also to vessels and edematous or scar tissue, for which MRI may not be specific enough [5]. On the other side, meningiomas, PitNET and SBPGL often feature an intraosseous growth pattern, which is challenging to define because of its lower contrast uptake compared to the soft tissue tumor component and hyperintense physiologic T1 signal of normal bone and marrow. For both of these scenarios, molecular imaging appears to be an optimal strategy to increase both sensitivity and specificity. All three tumor types commonly feature the overexpression of somatostatin subtype receptors 2 (SSTR2), which can be visualized with [^68^Ga]Ga-DOTA-SSTR PET radiotracers. The integration of [^68^Ga]Ga-DOTA-SSTR PET scans combines anatomical and functional information, allowing for a more accurate identification and delineation of the target volume, resulting in higher conformality of the treatment [6]. The goal of the present study is to evaluate the impact of adding PET/CT to CT and MRI imaging for clinical target volume (CTV) definition for proton therapy treatment planning for benign skull base tumors with SSTR2 receptor expression.

## 2. Materials and Methods

### 2.1. Patient Cohort

A total of 47 patients, of whom 30 had meningioma, 12 had PitNET/pituitary adenoma and 5 patients had SBPGL, were included in this study. The patients were treated with definitive PBT at the MedAustron Ion Therapy Center between August 2018 and February 2022. A histological diagnosis was not considered mandatory. In case of no prior biopsy, a clinical diagnosis involving clinical or endocrinological findings, an MRI and DOTA features for either meningioma, PitNET or SBPGL was considered sufficient. There was no limitation on the number of surgeries received, but all patients had to have a macroscopic tumor reported in the treatment planning MRI. The characteristics of the patient cohort are displayed in Table 1.

### 2.2. Imaging Protocols

Postoperative MRIs for proton therapy planning were performed according to the unified protocol of our institution. The following sequences were acquired using a Philips Ingenia 3T with a standard head coil: T1-weighted (3D mode, 768 × 768 px resolution, 4 mm slice thickness), T2-weighted (3D, 512 × 512, 1.9 mm), T1-weighted with contrast medium (3D, 864 × 864, 0.95 mm), fluid-attenuated inversion recovery (FLAIR, 560 × 560, 4 mm), diffusion-weighted images (DWI) in turbo-spin echo (b = 0–1000 s/m^2^), and apparent diffusion coefficient (ADC, both 256 × 256, 4 mm).

PET/CT studies were performed at least 6 weeks after the last surgery at an external nuclear medicine department with one of the following SSTR2-avid radiotracers: [^68^Ga]Ga-DOTATOC, [^68^Ga]Ga-DOTANOC or [^68^Ga]Ga-DOTATATE; the choice of radiotracer used was specific to each external nuclear medicine department. All radiotracers were generator-produced; no specific calibration for this study was performed, but all institutions follow their own internal quality control protocols, where the scanners are periodically tested against reference probes to ensure consistent readouts. After fasting for at least 6 h, the patients were intravenously administered 75–273 MBq of a tracer activity. After a resting period of up to 60 min, acquisition of low-dose CT and, subsequently, attenuation-corrected PET within 4 to 45 min (according to protocols of external institutions) of the head followed. The maximum standard uptake values (SUV_max_) and maximum SUV-to-background ratio, defined as the ratio between the SUV_max_ of the tumor lesion and the SUV_max_ of a representative region of interest (ROI) placed in the adjacent meninges or soft tissue, were recorded using IntelliSpace 8 software (Philips, Eindhoven, The Netherlands).

### 2.3. Study Design

Four independent observers were instructed to contour the macroscopic tumor volume on planning MRI. After the addition of information from the DOTA PET/CT images, they were asked to revise their original volumes. The observers are senior radiation oncologists with experience in CNS and BoS tumors. All of them were accustomed to using PET/CT for assistance in target delineation for BoS and CNS tumors, as this is a common practice at the institution where this study was performed. The observers had access to the anonymized clinical information including preoperative scans, as well as to in-built quantitative PET/CT parameter (SUV) assessment tools. There were no specific guidelines for the image interpretation introduced. The observers used their best judgement backed by experience.

The statistical methods used in this study followed the protocol published in [7]. The MRI images, including at least one of the following sequences, T1-weighted (3D), T2-weighted (3D), T1-weighted with contrast medium (3D) and fluid-attenuated inversion recovery (FLAIR), were uploaded to the treatment planning system RayStation 11B (RaySearch Labs, Stockholm, Sweden) and appended to the anonymized patient cases with rigid registration matched on the skull. Planning CT scans were used to set up a frame of reference and ensure that no shifts occurred during the MRI and PET fusion process. These shifts could affect contour interpretation and should be avoided. The correctness of the image matching was reviewed by medical physicists in treatment planning before handing the cases over to the observers.

The delineation process was performed in two rounds. First, the observers were requested to contour the gross tumor volume based on the preoperative images and the standard planning images (MRI, CT). After the completion, the contours were locked for further editing. A copy of the contoured volumes was created for the second delineation round that included analysis of the PET-CT imaging dataset. After that, the observers could review their contours in the context of the added PET information and adapt the volumes if required. All patient cases were anonymized prior to this study beginning, and the observers were blinded to the volumes of other participants.

### 2.4. Proton Treatment

The CTVs of the meningioma, PitNET and SBPGL cases were defined based on clinical information and serial images since diagnosis for planning CT, MRI and PET/CT (reference tumor volume). To account for setup inaccuracy and intra-fractional motion, the planning target volume (PTV) was defined as CTV expanded by 3 mm in all directions. The prescribed dose of 54 Gy was the same for all patients. The dose was applied to PTV in 27 fractions (2 Gy per fraction). The relative biological efficacy (RBE-value) of 1.1 was used for treatment with protons. The required target volume coverage standard was to achieve a median dose to PTV equal to the prescribed dose, and 100% of target volumes were covered in respective 95% isodoses [8]. According to the internal and international quality assurance standards, target volumes (CTV as well as PTV) and proton treatment plans were reviewed on internal medical boards prior to the treatment. Proton beam therapy (PBT) was delivered by applying 1 fraction per day, on average, 5 days per week. Follow-up evaluations included clinical visits and MRI at three, six, and twelve months after treatment completion, as well as annually (+/−3 months). 

### 2.5. Statistical Methods

To assess conformality between volumes, conformality indices (CIs) were calculated as described in [6]. These were defined as the ratio between intersection (common volume) and union (summary volume), which for hypothetical, ideally conforming volumes would be equal to unity. The respective volumes were created by the treatment planning system *Raystation 11B* using the in-built algebra function. CIs were calculated for MRI-only and PET/CT-assisted series between the following:

- Each possible pair of observers;
- Each observer and reference volume;
- The intersection between the volumes of all participants and the reference volumes.

The statistical calculations were performed using Stata BE 18 (StatSoft, College Station, TX, USA). The analysis of differences between the series was assessed via a non-parametric Wilcoxon matched-pairs signed-rank test. The test was two-sided, and results with *p*-values below 0.05 were considered statistically significant. The statistical analysis was performed for the whole patient cohort, as well as for the tumor types separately.

## 3. Results

**Figure 1:**
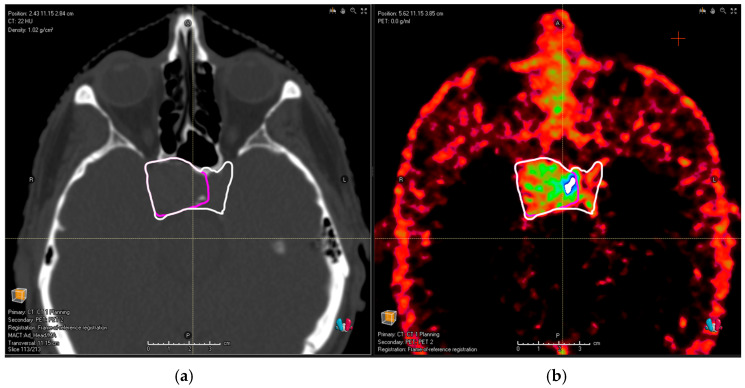
(**a**) Planning the CT image of a patient with pituitary adenoma. Pink contour shows common MRI volume; after the addition of PET/CT, (**b**) the observers identified an infiltration in the left cavernous sinus (white contour).

**Figure 2:**
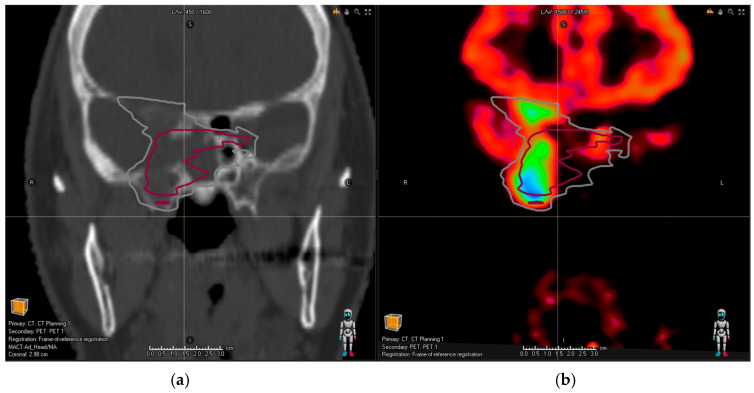
Planning CT image (**a**) of a patient with an aggressive pituitary adenoma. Red contour shows the common MRI volume of all observers; (**b**) the addition of PET/CT revealed further bone infiltration into frontobasis (difficult to identify based on structural imaging alone as the patient had co-existing osteoporosis)—the gray contour representing common PET/CT and MRI volume takes this finding into account.

The tumors were tracer-avid in all patients except for one with meningioma. The single case without tracer binding in PET/CT (SUV_max_ = 2.0, contrast ratio ~1:1) was not included in the further analysis. The median SUV_max_ value is 13.5 for meningiomas (range 6.6–31.2), 15.94 (range 2.5–21.62) for PitNET and 33.9 (range 12.6–133.3) for SBPGL. The corresponding median tumor-to-background ratios were 8.2 to 1 (3.7:1–20.8:1), 11 to 1 (2:1–24:1) and 38 to 1 (13:1–133:1) for meningiomas, PitNET and SBPGL, respectively. In total, we identified eight cases in which all four observers omitted parts of the tumor volume in the MRI-based contouring and identified them properly in the PET/CT-assisted series. In four cases of meningioma tumor, the omitted lesions were independent tumor nodules and, therefore, did not have an impact on the generated treatment plan. In two cases of meningioma and two cases of PitNET, the omitted tumor volume was in direct proximity or in continuity with the tumor volume but extending outside of the PTV margin. This specifically included the following:

- Meningioma with infiltration along the rectus medialis muscle;
- Meningioma with intraosseous extension in the contralateral sphenoid;
- PitNET with the bilateral involvement of cavernous sinus (as depicted in Figure 1);
- Extensive bone infiltration in the frontobasis area from a case of an aggressive PitNET (as depicted in Figure 2).

In all these cases, the new tumor volume first identified with the aid of DOTA PET/CT should be considered relevant for the proton treatment plan. There was no relevant tumor volume missed by observers in MRI compared to PET/CT for glomus tumors. Figure 1a shows the planning CT image of a patient with pituitary adenoma; Figure 2a shows the planning CT image of a patient with an aggressive pituitary adenoma.

There was a significant trend towards increases in the volumes contoured when using MRI and PET/CT, compared to MRI alone (Z = −4.53, *p* < 0.001). Specifically, this concerned 24 out of 29 meningiomas, all cases of PitNET and 4 out of 5 SBPGL cases. The intersection volumes were, on average, 1.77 cm^3^ (+20.2%), 1.84 cm^3^ (+6.7%) and 0.32 cm^3^ (+5.1%) bigger in PET-assisted series for meningiomas, SBPGL and PitNET compared to standard CT/MRI based contouring, respectively.

Regarding the median CI values between observer pairs, in most tumors, we identified a higher CI index in the PET/CT-assisted series (31/46 cases, z = −2.59, *p* = 0.01) corresponding to a better concordance between the observers. Separate analyses for tumor subgroups revealed certain differences between them. Especially for meningiomas, higher CI values in PET-assisted contouring series compared to MRI-only-based target definition was statistically significant (23/29 cases, z = −3.43, *p* = 0.0006). This was similar for SBGDL cases: four out of five cases had higher CIs when using PET/CT; yet, most likely due to small sample size, this trend did not reach statistical significance (z = −1.4, *p* = 0.14). The median CI relative difference was +4.4% in favor of PET/CT-assisted contours. The analysis of PitNET cases revealed a statistically significant trend in opposite direction: 9 out of 12 cases (z = 2.04, *p* = 0.04) had better median CI values for MRI-only-assisted series with a median CI difference of 1.1%. 

In a corresponding analysis of conformity between observer and reference target volumes, the results were only significant for meningiomas: in 22/29 cases, the median CI were higher (z = −3.71, *p* = 0.0002, median difference: +4.8%). For other tumors, the trends observed were analogous to the ones assessed between observers: three out of five cases of SBGDL had higher CI (z = −1.2, *p* = 0.22) in the PET-assisted series, whereas more cases of PitNET/pituitary adenomas had higher median CIs when contoured using MRI only (7/11, z = 0.9, *p* = 0.32). The median CI differences were lower than in comparison between observer pairs: +0.4% and −2.9% for SBPGL and PitNET, respectively.

No clinical factors contributing to the risk of omitting relevant tumor volume have been identified in logistic regression analysis. The median follow-up for all patients was 50 months (range 18–73). The 4-year actuarial overall survival was 95.4% (95%CI: 83.9–98.8%) and the local control was 97.7% (95%CI: 84.9–99.7%). One patient died of COVID-19; another one died shortly following proton therapy for PitNET/pituitary adenoma due to underlying comorbidities (diabetes, hypertension, cardiovascular disorder). During the follow-up period, one meningioma patient had fast-growing local infield failure, leading to a consecutive fatal hemorrhage 22 months after treatment completion.

## 4. Discussion

Radiotherapy plays a pivotal role in the management of a locally aggressive sub-population of benign skull base tumors that are not suitable for surgery. For this group of patients, radiotherapy, especially proton therapy, offers effective tumor control while minimizing therapy-related toxicity and preserving quality of life. Based on high dose falloff within millimeters, proton therapy is remarkably precise, and target definition becomes crucial. As precision medicine cannot exist without precision imaging, PET imaging has revolutionized the field of target definition in radiation therapy by offering additional valuable information to tumor delineation, particularly for patients who had already undergone previous treatments, like surgery or for tumors with locally aggressive growth, including intraosseous growth pattern or the infiltration of nearby structures [6,9].

We present an interobserver study comparing standard CT/MRI-only for target delineation versus standard and [^68^Ga]Ga-DOTA PET/CT-assisted series for benign skull base tumors with SSTR2 receptor expression in a cohort of 47 patients. Independent of the histology, the interobserver variability was reduced due to additional information from PET, resulting in a homogeneous target definition. The analysis of Rini et al. of a mixed group of SSTR-overexpressing tumors of the head and neck showed, similarly to our results, that in contrast to MRI imaging alone, somatostatin receptor–PET can detect additional ectopic lesions, as well as tumor parts infiltrating vascular structures such as cavernous sinus [10]. 

Meningiomas are the most common CNS tumors, and the majority are regarded as benign. However, a subset of patients presents with complex tumors involving several anatomic compartments of the skull base in whom gross total resection is not safely possible. Proton therapy is applied as a definitive treatment for primary or recurrent disease or postoperatively following subtotal resection [11]. In this context, [^68^Ga]Ga-DOTA-SSTR PET/CT is playing a major role in treatment planning and target definition [12]. The results for the meningioma cohort within the present study are in concordance with the current literature in terms of better agreement between observers, resulting in more consensual delineation and accurate target definition when integrating PET/CT into standard CT and MR. This finding underlines the importance of integrating PET/CT into target definition for skull base meningioma radiotherapy planning [13,14,15]. 

PitNETs and pituitary adenomas are histologically benign tumors with a good prognosis. Local control after surgical interventions can be challenging due to their complex locations and the limitations of the surgical techniques. Proton radiation has been reported to have excellent outcomes, and this highly conformal technique is also expected to benefit from the addition of PET/CT to structural imaging for delineation [16]. Wang and colleagues used PET/MRI and a dual-tracer setup ([^18^F]F-FDG and [^68^Ga]Ga-DOTA) to identify PitNET/adenoma recurrences and the extent of primary disease [17]. They noted a unanimous pattern of tracer accumulation: FDG uptake was higher than in normal pituitary parenchyma and [^68^Ga]Ga-DOTA binding was lower. We consider this phenomenon to be responsible for the paradox situation of the decreased confidence of observers in our study for this subgroup only after PET/CT was added. The clear threshold to differentiate normal pituitary from the tumor is not well defined, leaving room for variation connected to individual judgement and experience. It is worth mentioning that these discrepancies rarely cause a clinical effect, as a generous CTV involving a normal pituitary gland is added to GTV at our institution. In light of identifying new relevant disease extent in 2 out of 12 cases, the addition of PET/CT to MRI should still be considered positive.

Skull base paragangliomas are rare neuroendocrine tumors derived from the parasympathetic nervous system of the carotid body, glomus vagale, glomus jugulare or glomus tympanicum regions. These tumors have been historically treated with surgery, but due to surgery-associated risk of injury to nearby vascular structures and cranial nerves, a trend towards a more conservative management approach is observed, with an increasing proportion of patients treated with radiotherapy or considered for watchful waiting [3]. Similarly, for skull base meningiomas, SBPGLs can extend into adjacent structures, such as the temporal bone or intracranial compartments. The challenge in target delineation is to determine the correct tumor volume while protecting nearby organs at risk. Somatostatin receptor–PET has proven to be beneficial in detecting additional lesions and more extensive disease than contrast-enhanced MR imaging alone [10,18].

Astner et al. published, in [19], a phantom study on the assessment of tumor volumes in SBPGLs using Gluc-Lys [^18^F]-TOCA PET in comparison to MRI. Especially in recurrent tumors, the target definition determined by PET was significantly more homogeneous and smaller compared to MRI only. The volumetric increase in MRI is explained by the authors by a strong signal enhancement on MRI due to scar tissue; DOTA PET/CT is free of this limitation. In the present study, an increase in target volume definition was observed when incorporating the PET information. In our report, the change concerns common and not individual volumes; therefore, they are not comparable directly. On the other hand, we attribute this difference to the study setup: there were four observers in our study versus two in [19]; a greater number of observers contribute to a smaller intersection volume, which will increase with the confidence of observers due to added PET/CT information. Therefore, an increase in PET/CT absolute volumes is most likely an indirect representation of better concordance between observers. Regarding the direct assessment of interobserver variability, a trend was observed in our study towards better interobserver concordance with PET/CT, but in this subgroup, the result was not statistically significant, likely due to the small sample size.

We would like to point out some limitations of this study. First of all, statistical analysis was performed on separated tumor groups of a relatively small group size, namely the PitNET and SBPGL cohorts. This affected the statistical significance of the findings. To make conclusions that are more robust and reproducible between institutions, studies with larger group sizes would be advantageous. Secondly, our aim was to provide practical conclusions for the real-life clinical application of PET/CT for PBT planning. In this study, PET/CT scans were taken in multiple external institutions, and more than one tracer type was used. The absence of detailed guidelines on how to interpret MRI and PET/CT images, as well as no real-time corrective actions being taken towards observers, might have led to some biases in the results. We are, however, considering this as a common situation in everyday clinical practice, in particular in proton therapy centers without a proprietary nuclear medicine department. Being able—with board-discussed contours considered as a reference—to correctly delineate complex tumors despite these biases is an important added value of PET/CT in radiation oncology. The increase in conformality observed in our study confirms this advantage, and the interpretation of molecular imaging should rather be based on more standardized experimental setups, most optimally with an additional means of validation such as pathologic assessment.

## 5. Conclusions

In conclusion, the integration of [^68^Ga]Ga-DOTA-SSTR PET information holds promise for enhancing target delineation in radiotherapy for benign skull base tumors. Its integration into treatment planning provides valuable information that complements conventional CT and MRI, resulting in more accurate and personalized treatment strategies in meningioma and PitNET. Continued research efforts aimed at refining PET imaging techniques and addressing existing limitations are essential for further optimizing its utility in the management of SBPGL.

## Figures and Tables

**Table 1:** Patient characteristics. PitNET is pituitary neuroendocrine tumor/pituitary adenoma; SBPGL is skull base paraganglioma. “All observers” stands for the intersection between all observers.

Variable (Unit)	No. of Patients (%)	Median (Range)
Tumor type		
Meningioma	30 (63.8%)	
PitNET	12 (25.5%)	
SBPGL	5 (10.7%)	
Sex		
Female	35 (74.5%)	
Male	12 (25.5%)	
Age (y)		54 (21–81)
No. of surgeries		
1	36 (76.6%)	
>1	11 (23.4%)	
Reference GTV Volume (cc)		8.6 (3.06–89.7)
Meningioma		14.02 (3.89–67.2)
PitNET		7.0 (3.06–89.7)
SBPGL		19.15 (3.5–77.5)

## Data Availability

These data contain patient-related medical information. They are confidential and only available to certified trial auditors.
